# Towards a Ubiquitous User Model for Profile Sharing and Reuse

**DOI:** 10.3390/s121013249

**Published:** 2012-09-28

**Authors:** Maria de Lourdes Martinez-Villaseñor, Miguel Gonzalez-Mendoza, Neil Hernandez-Gress

**Affiliations:** 1 Universidad Panamericana Campus México, Augusto Rodin 498, Col. Insurgentes-Mixcoac, Mexico City, 03920, Mexico; 2 Tecnológico de Monterrey, Campus Estado de México Carr. Lago de Guadalupe, Km 3.5, Col. Margarita Maza de Juárez, Atizapán de Zaragoza, State of Mexico, 52926, Mexico; E-Mails: mgonza@itesm.mx (M.G.-M.); ngress@itesm.mx (N.H.-G.)

**Keywords:** ubiquitous user model, interoperability, web service, overweight and obesity

## Abstract

People interact with systems and applications through several devices and are willing to share information about preferences, interests and characteristics. Social networking profiles, data from advanced sensors attached to personal gadgets, and semantic web technologies such as FOAF and microformats are valuable sources of personal information that could provide a fair understanding of the user, but profile information is scattered over different user models. Some researchers in the ubiquitous user modeling community envision the need to share user model's information from heterogeneous sources. In this paper, we address the syntactic and semantic heterogeneity of user models in order to enable user modeling interoperability. We present a dynamic user profile structure based in Simple Knowledge Organization for the Web (SKOS) to provide knowledge representation for ubiquitous user model. We propose a two-tier matching strategy for concept schemas alignment to enable user modeling interoperability. Our proposal is proved in the application scenario of sharing and reusing data in order to deal with overweight and obesity.

## 1. Introduction

Communications and technology have become an important part of day-to-day activities. People interact with systems and applications through several devices and are willing to share information about preferences, interest and characteristics. Each user has different needs, preferences and interests that can change in an instant. Therefore, personalizing and adapting product or service that fulfills user's present need adequately is a challenging task, but it is a must to allow the growth and penetration of ubiquitous applications. Sometimes, the user has to settle for stereotyped products and services. If the user does not get what he/she wants or he/she has to spend more time searching for a suitable alternative to his/her needs, or will stop using the application.

In order to change the behavior of devices, systems and applications, and to achieve a more personalized delivery of product and services, it is necessary to model the user's profile [1,2]. Currently, many service providers have user-adaptive systems and are trying to model the user for adaptation and customization, but these efforts are frequently isolated and repeated by each supplier. In user-centered computing environments, the key for personalization is to collect user's information and construct a ubiquitous user model [3] that represents the user's characteristics, preferences, interests, intentions and needs as accurate as possible. Service providers build user models using different well known techniques [4], collect explicit information provided by the user, implicit information obtained from embedding Human-Machine Interaction (HMI) technologies, applications and devices and indirect information obtained from advanced sensors and complementary systems. The computational effort of building a user model is repeated across different platforms, applications and systems in various domains. The user has to invest time in setting parameters of devices and services repeatedly, which leads to overabundance of information, inconsistency, redundancy and repeated configurations. The information of the user is scattered over different user models, so each application has just a narrow understanding of the user, given by some features of the user profile.

Some researchers in the user modeling community [3,5–7] envision the need to share user models' information between applications in order to enhance and integrate the knowledge of the user. With a better understanding of the user, adaptive systems can provide better service, content and interface personalization and adaptation. Sharing and reusing information between models can bring advantages for profile providers and profile consumers; it helps dealing with the above mentioned problems, provides enrichment for the user models and frees the user from the need to repeat the configuration process, but gathering distributed user information from heterogeneous sources implies interoperability problems [7]. The lack of interoperability between adaptive systems is due to the syntactic and semantic heterogeneity of the different user models.

Several techniques have been proposed to deal with the lack of interoperability, and support user modeling for cross-system personalization [5,8,9]. The two mayor approaches for user modeling interoperability are the definition of standard ontologies that provide a common understanding among multiple systems or a conversion approach using mediation techniques to provide mappings between the different user models.

The development of a standard ontology to provide a static common understanding for multi-application personalization is not a feasible solution, it has become clear in recent years, that the user model representation must be able to evolve over time [8]. The ubiquitous user model must be flexible to allow personalization of frequently used and new applications in different possible contexts.

Berkovsky [6] gives a definition of mediation to introduce cross-system personalization using techniques that match and integrate user models. The main obstacle of mediation approaches is overcoming the heterogeneity of the user modeling data [10]. In [10], the authors suggest bridging the gaps between both approaches. Among other future research directions, they recommend the use of domain thesauri and ontologies and in particular the use of WordNet for identifying synonyms and enhance reasoning and mediation of user modeling data. They also see the need to develop flexible mechanisms that will allow applying ontologies in different domains, situations and constrains.

In this paper, we address the interoperability problem between heterogeneous sources and the evolutivity problem trying to overcome the limitations of both approaches. We present a dynamic user profile structure based in Simple Knowledge Organization for the Web (SKOS) to provide knowledge representation for ubiquitous user model, and we propose a two-tier matching strategy for concept schemas alignment to enable user modeling interoperability. The process of concept alignment provides articulation between profile suppliers and consumers establishing the mappings from individual concepts of the sources with the corresponding ubiquitous user model concepts. This process of concept alignment consists in two steps, an element level matching and a structure level matching. In addition to enable interoperability, this process determines the evolution in time of the ubiquitous user model deciding when a new concept must be added.

The main contributions of our approach, the semantic representation of the ubiquitous user model and the process of concept alignment, will allow dealing with syntactic and semantic heterogeneity with the least intervention of profile and provide a semantic representation for the user model that adapts itself to new profile suppliers and consumers. Our proposed solution provides flexibility overcoming the limitations of a static user model ontology, and the semantic schema matching that defines semantic concept mappings is done automatically unlike other authors approaches that establish the mappings manually or semi automatically. We present a ubiquitous user model to enhance the understanding of the user and provide more proactive and personalized services through sharing and reusing profile information with semantic web technologies. We give an example (Section 5.2) to illustrate how reusing and sharing profile information from heterogeneous sources can be done with the mediation of our ubiquitous user model and the process of concept alignment. This example considers the scenario using information from different user profiles to deal with overweight and obesity. In this scenario, static, semi-static and dynamic information is necessary, and heterogeneous sources are involved: social network applications, personal health records, specialized health sites and web applications, FOAF and personal devices with attached sensors. We specifically reuse the ubiquitous user model information to automatically personalize a web service populating its input parameters.

For our experimentation, the ubiquitous user modeling ontology based in SKOS was set up with real data of Facebook, FOAF and one profile of a specialized web application to monitor person's diet and physical activity of one user. We gathered information about training sessions with Polar devices and automatically established the pertinent mappings to enable the interoperability with the mentioned sources. The process of concept alignment also identified useful concepts from the user model that can satisfy a provider's web service input parameters requirements and personalize its instantiation.

The ubiquitous user model is part of an ongoing project to develop a user-adaptive system based on a distributed architecture to exchange, merge and share profile information contained in existing heterogeneous user models.

The paper is organized as follows: in Section 2, we present an overview of the main characteristics of the ubiquitous user model and heterogeneous sources considered. In Section 3 we review the existing techniques related to the construction of a ubiquitous user model. Our proposal is explained in detail in Section 4. The application scenario, dealing with overweight and obesity, is presented in Section 5. Demonstration of the functionalities and their results are shown in Section 6. We compare our approach with related work in Section 7 and conclude in Section 8.

## 2. Ubiquitous User Modeling stakeholders

There has been a great evolution of user modeling and adaptation from desktop paradigm to ubiquitous computing [11]. There are user models built in service provider's side described in [4] and models constructed by the user for personalization. In [3] it is stated that “ubiquitous user modeling can be differentiated from generic user modeling by the three additional concepts: ongoing modeling, ongoing sharing and ongoing exploitation”. We are considering a ubiquitous user model that is application and system independent. Our user model must not only be independent of applications, devices and systems but must also allow the interoperability between distributed user profiles. It must allow ongoing sharing and reusing between heterogeneous sources in a changing environment. In recent years it has become clear that the user model representation must be able to evolve over time [8] due to the great deal of syntactical and structural differences between user models. It is practically impossible to develop a static commonly accepted ontology of the user modeling interoperability domain.A process of sharing and exchanging data of user profiles is necessary in order to achieve cooperation between systems and applications. Ubiquitous user modeling stakeholders play two main roles: profile information supplier, and profile information consumer. These roles can be played by different agents that can act in one or both roles simultaneously. Currently, a user has many profiles in different devices, applications and systems. Many personal gadgets have advanced sensors that gather data about the user on real time. We choose to work with social networks profiles, FOAF [12] and microformats, and personal devices with sensors. Social networking sites in particular, have become very successful and can be considered as valuable sources of personal information in order to build a user model. According to Nielsen, in a study published on March 2011 [13], people in the U.S. spent more time on Facebook than in any other website. Social network profiles can enrich user models from other providers too. These applications can be profile information suppliers and consumers at the same time.

Semantic Web technologies such as FOAF and microformats represent an important step forward in terms of the Web's evolution. They provide a way to embed machine readable structured data into web pages for expressing user personal information [14]. We selected FOAF as an important profile supplier as it is currently considered as one of the best populated ontologies and is extensively adopted to describe users and their relations [15]. So, RDF documents published using FOAF vocabulary are valuable sources for our user model.

Sensors attached to personal devices provide contextual information mainly about the user's. location, movement, distance and identity. Users' behaviour can also be collected through mobile devices such as smart phones that have built-in sensors [16]. Sensor devices deliver data relevant to healthcare, homeland security and tactical training that help to identify and handle situations on real-time [17]. In [18] the authors state that “to progress towards a full harmonization between HMI systems and Sensor Web advances are needed in two fundamental areas: the integration and accessibility of a growing number of heterogeneous sensor data into HMI systems, and new mechanisms that allow sharing real-world information provided by users of connected objects into the Sensor Web or the Semantic Sensor Web”. In summary, profile information suppliers provide together valuable static, semi-static and dynamic information about the user.

The major web sites are transformed into web services to really expose their content for reuse. Amazon is a pioneer in web services strategy for electronic commerce, and Yahoo has also began the transformation into structured information. In this work, we consider web services as profile information consumers as we seek to use the ubiquitous user model to adapt and personalize web services. Web services are, nowadays, emergent technologies to consume information on the web, but in order to improve their usability, personalization is needed. Web services' personalization has been done in service discovery, selection and composition [19], and in the step of service instantiation. The latter means “adapt services to users by identifying useful parts of their profile that satisfy service requirements” [20]. We selected Web services as profile consumers because the user model can be used to customize different steps of interaction with services. User profile information from heterogeneous sources can be reused as hard constrains in the steps of service discovery, selection and composition, and/or as soft constrains identifying useful concepts from the user model that can satisfy web service input parameters requirements. We show an example of the latter process in Subsection 6.2 with a SOAP request which is a Training Peaks Web Service, but this process can also work for RESTful Web Services.

## 3. Related Work

In the user modeling community, two approaches were proposed [8–10,21] for achieving syntactic and semantic interoperability between heterogeneous sources: a shared format approach *vs*. a conversion approach. The former imposes the use of a shared syntax and semantics to represent the user model, and the latter employs algorithms and techniques to convert the syntax and semantics of the user data and enable the interoperability between two systems. In *standardization-based* user modeling approach sharing and reusing user models is easier, but it requires all systems to adhere to a standardized ontology and representation. *Mediation-based* approaches deal with user modeling information sharing in a practical way, but they require a large number of transfer mechanisms in the same or across domain. “They main obstacle for materializing the mediation ideas is overcoming the heterogeneity of the user modeling data” [10].

### 3.1. Standardization-Based User Modeling

One of first attempts towards standardization is Doppelgänger [22], a generic user modeling system that gathers data about the user from sensors and derives a general model for the user. This system doesn't really define a formal structure for data exchange, but established the importance of having a common data base about the user to increase applications abilities to personalize themselves. Concerning standardized modeling approaches based on ontologies we can mention [3,23–27]. UM [23] is a toolkit for user modeling and a good example of how to build a generic user model from the accretion of evidence of knowledge, beliefs, preferences and other user characteristics without interpretation. UM toolkit allowed cooperative modeling and was designed to do user modeling tasks in concrete applications like coaching systems and movie recommenders. The authors describe in [24] an architecture for distributed personalization better suited for ubiquitous computing environments. This architecture presented a central user model and several partial user models intended for different applications. A complete framework to allow exchange and the semantic integration of partial user models is presented in [3]. The general user model ontology GUMO contains information of user characteristics, emotional state and other aspects of user personality. In [3], the author showed how to gather explicit, implicit user data and context data to construct a situational model that allows adaptation and personalization according to user's situation. The model is almost static and doesn't provide a way to describe several user profiles. It provides a vocabulary but not a structure to support several profiles. It is clear that developing a commonly accepted ontology to deal with sparseness of data and heterogeneity of sources in a multi-application environment is not a feasible solution.

### 3.2. Mediation-Based User Modeling

Attempts of bridging user modeling in various systems require conversion of user modeling data between various representation, applications and domains, and exploiting semantically-enhanced knowledge bases [10]. These has been done using mapping techniques of user models [6,28–31], and recently using machine learning techniques [10].

Vassileva *et al.* [28] used mediation techniques based in multi-agent technologies. In their approach the agents map help requests to possible service providers. Berkovsky *et al.* [6] provided mediation and integration in recommender system domain. Houben *et al.* [29] developed a Generic User model Component (GUC) using Semantic Web technologies to provide mediation between web applications. A human designer constructs a rule-based mapping schema. Mappings are not defined automatically. In 2005, Lorenz [30] proposed an open architecture for sharing partial user models. They provided a well-defined conceptual basis of the conversion mechanism. More recently, Carmagnola *et al.* [7] presented a solution with high flexibility representing user models and providing semantic mapping of user data from heterogeneous sources. However, to take part in the interoperability process, every provider must comply with a standard format for exchange and maintain a sharable user model which includes fragments of it as RDF statements.

A semantic layer to improve mapping among application was presented in [31]. The authors showed a flexible user modeling system based on the concept of mapping among applications. They focus in management of user profile data propagation. Their G-Profile formalized the correct identification of mappings between applications; it is an abstract and flexible protocol.

Each profile provider builds a specific user model, but they do not easily comply with a standard format, vocabulary or technique to enable user modeling interoperability. On the other hand, mappings are defined at different granularity, and it is not always implemented by a fully automated process.

## 4. Outline of the Approach

Our user-adaptive system is a work-in-progress project based on a distributed architecture to share and reuse profile information contained in existing heterogeneous user models: social networking applications, personal health records, FOAF and microformats, and personal gadgets with or without sensors attached. We seek to obtain the following advantages when sharing and reusing information between user models:

- Relieve the user from repeatedly setting parameters in devices, applications and services which provokes inconsistency, redundancy and repeated configurations.
- Help dealing with “cold start problem” when a user is new to applications and services.
- Provide enrichment to the user models obtaining a better understanding of the user having a broader picture of user's characteristics, preferences and interest as well as user's current state.
- Reuse information as soft constrains identifying useful concepts from the user model that can satisfy web service input parameters requirements, in order to automatically personalize Web Service instantiation.

In this section, we present our progress in the construction and maintenance of a flexible ubiquitous user model that represents the knowledge of some commonly used profile suppliers/consumers. First, we design the user profile structure and decide which dimensions to consider. In order to make this design decisions, we: (i) determine the minimum requirements of our user profile reviewing some international initiatives towards standardization of user profile structure; (ii) analyse the nature of the information and its relevance in the ubiquitous user model; (iii) set up our user representation for some selected commonly used applications and devices. These concepts are presented in Section 4.1.

Secondly, an important design decision is the user modeling architecture that supports the flow of user modeling interoperability. The user modeling architecture determines the exchange patterns considered between profile suppliers and consumers and the resulting syntactic and semantic bridges needed to enable the flow of information. Traditionally there are two ways to approach this problem: a centralized model with which every actor establishes communication or a peer-to-peer communication distributed model. We defend the decision of adopting a centralized user model despite of the ubiquitous nature of the information in Section 4.2.

We briefly describe the architecture of the user-adaptive system that enables profile information sharing and reuse in Section 4.3.

Finally, we explain the alignment process to achieve interoperability between heterogeneous sources in Section 4.5. This process is crucial for the construction and maintenance of our ubiquitous user model; it enables interoperability and permits the evolution in time of the user profile structure.

We propose a flexible user model structure to minimize the redundancy and inconsistency of profile data. We developed the ubiquitous user model interoperability ontology to provide a share and common understanding between profile suppliers and consumers (Section 4.4). The main idea is to crumble the information extracted from the suppliers' profiles by concept and finding the semantic relations between each of them and most similar concept already stored in the user model. Once the relation is found, a link is established via semantic relation and mapping properties. A data model that provides us a common data model to enable data sharing across diverse applications is Simple Knowledge Organization System (SKOS) [32] and it can be used in combination with OWL as a formal knowledge representation language. SKOS is also a W3C recommendation since 2009.

The ubiquitous user model (u2m) is a classification scheme build using SKOS, which provides properties that specify hierarchical and associative semantic relations, and mapping properties, “used to state mapping (alignment) links between concepts in different concept schemes, where the links are inherent in the meaning of the linked concepts” [32] among other useful properties for our purpose. SKOS provides several properties that map concepts between different concept schemes, these are the most relevant properties taken into account for the alignment of our concepts. For asserting that two concepts have a similar meaning we can use the skos:exactMatch and skos:closeMatch properties. Two concepts from different concept schemes can also be mapped using properties that parallel semantic relations skos:broadMatch, skos:narrowMatch and skos:relatedMatch.

### 4.1. User Profile Structure

The task of building a flexible ubiquitous user model ontology that represents knowledge of all possible user profiles from providers and consumers actually used by a person and to come is very ambitious. As we described in the related work, developing a commonly agreed standard ontology for sharing and reusing information in heterogeneous sources for different domains is not a feasible solution. We build a ubiquitous user model that starts with a user profile structure representing generic settings according to recommended standards and most commonly used applications and devices. From this setting on, the ontology and therefore the ubiquitous user model evolves and changes the profile structure to adapt to new actors of the user modeling interoperability process.

With the purpose of defining the start-up user profile structure for our ubiquitous user model, we reviewed some international initiatives towards standardization of user profile structure; we analysed the nature of the information to determine its relevancy and permanence in the ubiquitous user model; we set up our user representation for some selected commonly used applications and devices.

We reviewed the following initiatives towards standardization of user profile structure: European Telecommunications Standards Institute (ETSI) [33], 3rd Generation Partnership Project (3GPP) GUP [34] and MAGNET Project [35].

With the above guides in mind, we established standard requirements for our user profile structure taking into account the recommendations relevant to our purposes.

- The user profile structure must at least consider information of basic profile with demographic data and general user data, device and service profiles, user preferences and interest. The profile should be flexible to be used in changing environments.
- Redundancy must be avoided. And if a specific data exists in one supplier profile, other profile actors considered in our system must be able to consume it or enrich its profile with it. (*Cfr.* ETSI Guideline 4.1.4.a).
- The user profile model provided must be directly usable by profile providers and consumers or for it to be translated. (*Cfr*. ETSI Guideline 4.1.5a).

**Figure 1.**
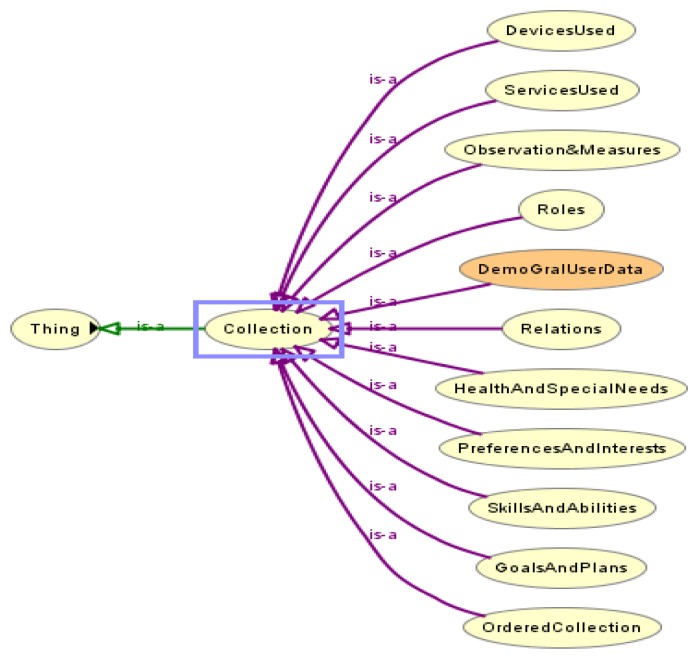
Ubiquitous user model collections.

According to the first request, the user profile structure contains concepts to define knowledge in the dimensions shown in Figure 1. Each of these dimensions is modeled with a corresponding skos:Collection of our skos:ConceptScheme in the ubiquitous user modeling ontology described in Section 4.4.

The profile structure is flexible because new concepts and sub collections can be added to these dimensions. Adding new dimensions dynamically is been considered.

If we analyse the nature of the information of user profiles, we find that some data are static like name or home town, some data are semi-static for example allergies or disabilities, and some data are changing constantly for instance current location or daily calorie intake. Every data is important for personalization and adaptation of one particular application in current user situation, but dynamic and semi-static data lose their validity in a period of time. The nature of the information influences the permanency and validity in the ubiquitous user model, but it is not determined by itself. For this reason we included static, semi-static and dynamic concepts in the start-up configuration of the ubiquitous user model.

In addition, we have to provide the ontology set up with representation of the profiles of most commonly used applications. We justified our selection of FOAF, Facebook and personal devices with sensors as profile suppliers and Web services as profile consumers in Section 2. We consider they are representative of most commonly used profiles and consumers. Personal Health Records contain valuable health information and will be included in the ubiquitous user model, but they are not widely adopted yet. We included one profile of a specialized web application to monitor person's diet and physical activity that we considered relevant for the application scenario and example described in Section 5.

Simple Knowledge Organization System (SKOS) provides us a common data model to enable data sharing across diverse applications and it is used in combination with OWL FULL as a formal knowledge representation language. Information extracted from the supplier's profile is crumbled by concept and organized in a SKOS concept scheme. Only one instance from each source with the most recent information extracted is stored in the U2MIO ontology and the ubiquitous user model concept scheme has just one concept of each similar attribute. Alignments between most similar concepts are determined in order to enable the interoperability with concept schemes of other sources. Once the relations are found, links are established via SKOS mapping properties. This approach differs from recent approaches [36] based on user profile management and selection that aggregate profiles and sub-profiles for each application, and select the best suited sub-profile for each situation related to a service. This causes redundancy when saving the same profile structure and information with minor changes several times.

### 4.2. Exchange Patterns and Processes

We identify the following exchange patterns to fulfill the goals described in Section 4.0. In the first exchange pattern, data from social network applications, devices and semantic web technologies like FOAF and microformats are harvested, interpreted and integrated to a ubiquitous user model. From this moment on, the user knowledge can be used to personalize web services or user model enrichment by the mediation of the ubiquitous user model (see Section 6.1).

**Figure 2.**
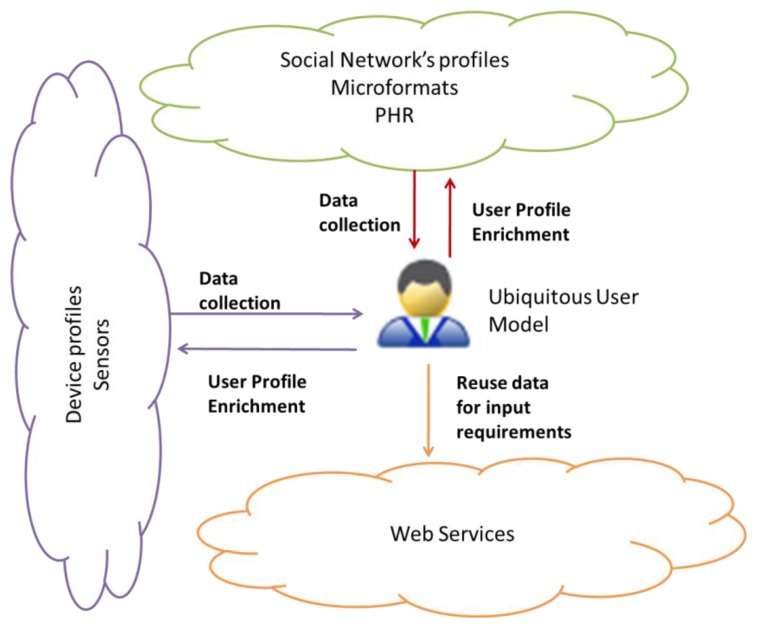
Exchange patterns between profile suppliers and consumers.

In the second exchange pattern, a profile supplier changes its role to profile consumer and reuse information of the ubiquitous user model to enrich its profile. A new application or system can take advantage of the knowledge acquired from the user also. Figure 2 shows these two exchange patterns. The case of Web Services is interesting because the system can reuse ubiquitous user model's data to populate Web Services' input requirements, and some providers' web services functionality permit the enrichment of some applications (see Section 6.2). To achieve the data exchange between these heterogeneous sources, it is necessary to establish communications and relate syntactically and semantically the concepts of the profiles with each other. The number of bridges between every two applications is from order of N^2^ if we choose a peer-to-peer communication. At syntactic level, this means speaking the same language and using the same format and protocol when communicating. From the semantic point of view, it entails sharing the same meaning, or finding a way to interpret and translate each concept.

We propose an approach establishing a central ubiquitous user model, and defining semantic mappings between the profile suppliers and consumers with the central model at concept level. From the exchange patterns in Figure 2, we determined that we need one semantic bridge from each supplier/consumer to the ubiquitous user model. These one-to one mappings at concept (attribute) level allow the interpretation of the information. The collection of user profile data and enrichment of user profiles has to take place according to the nature and conditions of each actor.

Given the nature of the heterogeneous profile suppliers and consumers, and the flexibility needed to add new actors, and the complexity of ubiquitous environment, an integrated user model is a better option. The number of bridges decreases from order of N^2^ between every two applications, to order of N between each application and a user model [5]. Nevertheless, it is still a difficult task to communicate and build an understanding between each supplier/consumer and the user model, without mentioning the design and implementation of the user model itself.

We try to benefit from the distribution of the profiles, but simplify the task of communication with a centralized user model. This implies great flexibility for adding new sources, but on the other hand, some choices on the data structure and means of translation to enable interoperability with the ubiquitous user model.

The actors (profile suppliers and consumers) will not interact with each other. Every exchange will be performed with the mediation of the ubiquitous user model. The required processes performed by the adaptive system are:

1. The construction of the ubiquitous user model by harvesting, interpreting and integrating data from the suppliers to the user model.
2. Update and maintenance of the ubiquitous user model.
3. The selection and extraction of personal data from the ubiquitous user model in order to personalize web services.
4. The selection and extraction of data from the ubiquitous user model to enrich new or existing user models.

### 4.3. Architecture for Profiles Exchange and Reuse

**Figure 3.**
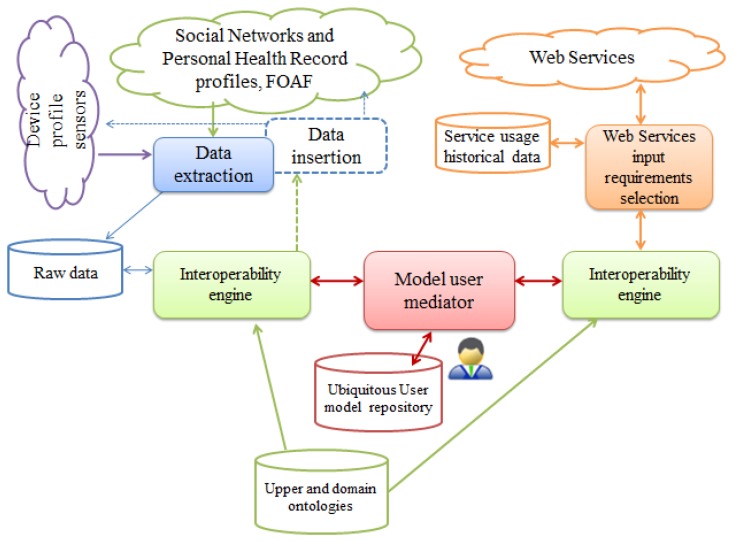
Architecture of adaptive system for profiles exchange and reuse.

The architecture of the adaptive Web-based system is shown in Figure 3. Each profile supplier has its format and data transfer particularities. The most common formats are XML, JSON and RDF. Each social network application has its own policies which we have to respect, and extraction and insertion APIs that are used to interact with the user profile. The system has to deal with each source transfer mechanisms. Sensor devices often need specialized hardware and/or software for data transfer. So each source has its own data extraction implementation.

The interoperability engine implements the process of concept alignment (described in Section 4.5) between the concepts of extracted documents and the concepts of the ubiquitous user model, and determines if instance transformations are needed. The alignment process determines semantic mappings providing articulation between the heterogeneous sources, and instance transformations enable interchangeability. The user model repository consists of ubiquitous user model interoperability ontology which provides semantic representation of the ubiquitous user model, and the profile instances. The U2MIO ontology (see Section 4.4) is stored in the ubiquitous user model repository and it contains the user model concept scheme, the concept schemes for each supplier/consumer with the most recent instance and semantic mappings to enable the interoperability.

This ontology will change in time depending on each user interaction. The model user mediator is responsible of manipulating the ontology according to the results provided by the interoperability engine. It adds the properties, classes and instances needed to articulate the concepts of the source document with the concepts of the ubiquitous user model. The interoperability engine performs similar operations to enable the semantic interoperability between the web services' input parameters and the ubiquitous user model concepts. It is important to notice that the ontology will be evolving over time in order to adapt its semantic representation to allow the user modeling interoperability with new profile suppliers and consumers. Finally, service usage historical data are collected for further mining in search of interesting usage patterns and identification of user intentions.

### 4.4. Ontology for Ubiquitous User Modeling Interoperability

In this section, we present a flexible ontology for ubiquitous user modeling interoperability (U2MIO) and its relevance for the user-adaptive system proposed.

The U2MIO ontology:

- Provides semantic support for user model overcoming differences between concepts at knowledge level.
- Represents a flexible user profile structure, with domain independency which provides the possibility for the ubiquitous user model to evolve during time.
- Provides representation for new profile suppliers and consumers that take part in the interoperability process without effort of the provider or consumer system.

The ontology reuses SKOS ontology and it can be seen as an aggregation of concept schemes each one representing a profile supplier or consumer, and a central ubiquitous user model concept scheme. Semantic mapping relations are established between each supplier/consumer concept scheme and the ubiquitous user model concept scheme at concept level by the process of concept alignment in order to enable interoperability between user models.

The ubiquitous user modeling ontology was set-up with Facebook, FOAF, and one profile of a specialized web application to monitor person's diet and physical activity of one user. This demands the design of four concept schemes, one for each profile provider and the ubiquitous user model concept scheme. Semantic mapping relations were established with SKOS properties. We used Protégé ontology Editor for the set-up process.

The ubiquitous user model concept scheme was set up with the basic skos:Collections shown in Figure 1 and their sub collections.

**Figure 4.**
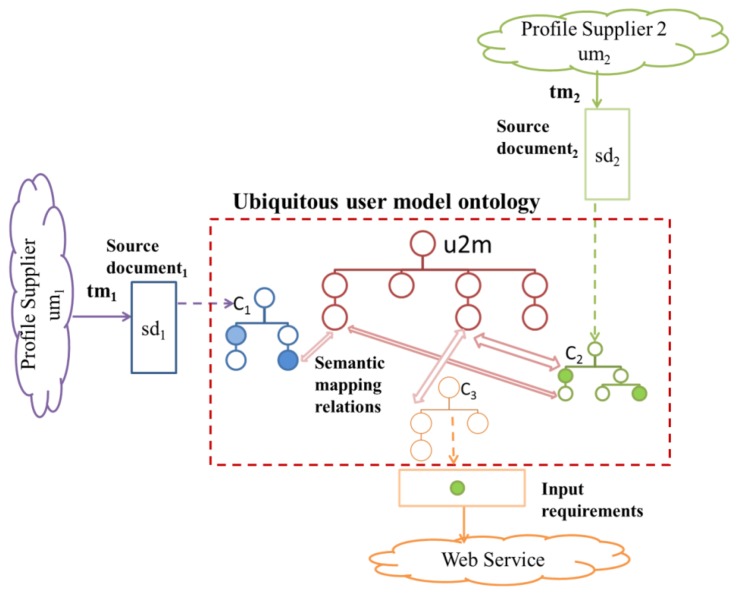
Interrelations between profile supplier/consumer and the ubiquitous user modeling ontology.

We briefly described the adaptive system architecture for profile exchanges (Section 4.3). We describe the interrelations between profile supplier/consumer and the ubiquitous user modeling ontology (U2MIO) in order to understand the relevance of the ontology and the process of concept alignment in the user modeling interoperability process (Figure 4).

In a multi-application environment we can find many profile suppliers. Profile suppliers have user models (*um*) and their own transfer mechanisms (*tm*). The process of data extraction deals with providers' transfer mechanisms to collect the profile data from the sources and obtain source documents (*sd*) in XML, JSON or RDF. If a profile supplier is new to the system, a corresponding concept scheme (*C*) must be designed and added to the ubiquitous user modeling ontology by the model user mediator. The interoperability engine implements the process of concept alignment that: (i) determines the semantic mapping relations at concept level from the source concept scheme (*C*) to the ubiquitous user model concept scheme (u2m); (ii) decides whether new concepts must be added to the ubiquitous user model concept scheme (u2m); (iii) decides whether new sub collections must be added to the ubiquitous user model concept scheme (u2m).

If a new profile consumer wants reuse profile information, it is also necessary to build the corresponding concept scheme and semantic mapping from the request in case of Web services or a XML, JSON or RDF schema. The process of concept alignment to allow interoperability in heterogeneous sources is described in the next section. The innovation of this approach is that the central ubiquitous user model is not static and that the interoperability is enabled at concept level. The ontology can evolve over time.

### 4.5. Process of Concept Alignment for Interoperability in Heterogeneous Sources

In order to integrate a ubiquitous user model gathering information from heterogeneous sources and be able to reuse this information, alignment between similar concepts must be done.

The alignment process is intended to achieve interoperability between a document written or translated to XML (named source) with the ubiquitous user model (named target). The ubiquitous user model (u2m) will provide an articulation between heterogeneous sources given the mappings from all individual concepts of the sources with the corresponding ubiquitous user model concepts. These mappings enable the interoperability between user models. The ubiquitous user model will provide articulation with Web Services' preferred values when they are requested.

The proposed matching strategy is similar than the one presented in [37], that integrates several methods in a three-tier matching strategy for predesigned schema elements. We propose a two-tier matching strategy. In this section, we present the formal problem statement and explain the schema matching techniques and alignment strategy used to determine the similarity and mappings between a given interoperability stakeholder and the ubiquitous user model concept schemes.

#### 4.5.1. Problem Statement

Following the work in [38] we define a mapping element as a triple: <*c_s_,c_t_,R*> where

- *c_s_* is a source concept expressed as skos:Concept from a source concept scheme *X*
- *c_t_* is a target concept expressed as skos:Concept from the ubiquitous user model scheme *U*
- R is a semantic relation (e.g., *equivalent* (=); *related* (⊆,⊇,⊓); *independent*: (⊥) holding between the entities *c_s_* and *c_t_*.

**Figure 5.**
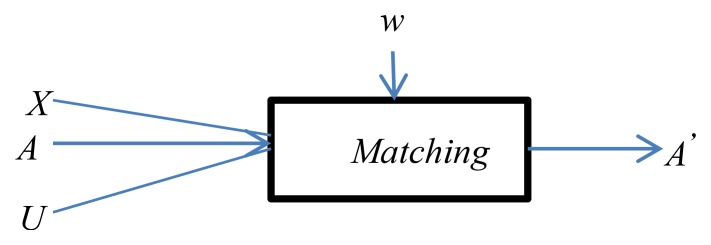
The matching process.

The matching operation determines the alignment (*A*′) for the pair of schemas *X* and *U* where *X* can be any concept scheme constructed from a profile supplier/consumer document and *U* will always be the ubiquitous user model concept scheme. See Figure 5.

*A* denotes a previous alignment for this pair of concept schemes (when available) which is to be completed or modified, *w* is an external resource used by the matching process (e.g., WordNet in our case).

Two basic elements are available to find mappings between concepts: concept labels and hierarchical structures. Concept values can be used in conflict resolution when a concept is consumed and to determine if further transformation is necessary for interchangeability.

We consider a concept scheme as *(C, H_C_, V_C_)* where C is a set of concepts arranged in a subsumption hierarchy *H_C_*. *V_C_* is the set of corresponding concept values if available.

Each concept *c_s_* in a set of concept source *C_S_* is defined by: a label *string1:string2* where *string1* is optional and *string2* is mandatory, and subclass relationships. When namespaces, other than default, are specified in the source document *string1* is used. The attribute or element identifier corresponds to *string2* and it is typically described as a simple or compound word in natural language. A subclass relationship sets up a link with other concepts in the source document. The hierarchical structure most be described as a skos:ConceptScheme.

A concept on the target side *C_T_* is described by set of labels included in the target skos:Concept consisting of lexical labeling, notation and documentation SKOS properties.

#### 4.5.2. Used Matching Techniques

In order to determine the similarity between a concept *c_s_* in *C_S_* and a concept *c_t_* in *C_T_* we use the Equation (1) which combines three matching techniques:

(1) $$\text{sim}\left(c_{s},c_{t}\right)=\max\left({\text{sim}}_{\text{Dice}}\left(c_{s},c_{t}\right),d_{\text{lcs}}\left(c_{s},c_{t}\right),{\text{sim}}_{\text{wordnet}}\left(c_{s},c_{t}\right)\right)\forall c_{s}\in C_{S},\forall c_{t}\in C_{T}$$

The inclusion of each of these three has a purpose. Similarity based in Dice coefficient [39] (*sim_Dice_*(*c_s_*, *c_t_*) ∈ [0,1]) has the purpose of finding if concept *c_s_* in *X* and a concept *c_t_* in *U* have a high lexical similarity. Dice coefficient is a simple and normalized similarity measure [40] that can show if the two concept labels are similar at string level. Equation 2 calculates the longest common substring distance similarity (*d_lcs_*(*c_s_*, *c_t_*) ∈ [0,1]) which tries to find if one label is subsumed in the other. The semantic similarity (*sim_wordnet_*(*c_s_*, *c_t_*) ∈ [0,1]) is based in Wu and Palmer path lengths method [41] using WordNet [42] as an external resource and tries to find the semantic similarity of the labels. Other languages similarity can be also included if the proper lexical resource is available:

(2) $$d_{\text{lcs}}\left(c_{s},c_{t}\right)=\frac{\text{Lenght}(\text{LCS}(c_{s},c_{t}))}{\min\left(\text{Lenght}(c_{s}\right),\text{Lenght}\left(c_{t}\right))}$$

The main methods used are shown in Table 1.

Finally, the highest of the three similarity measures is considered to determine relation *R* in the triple <*c_s_,c_t_,R*> of a mapping element according of the following criteria:

1. *Equivalent* (=): Two concept elements *c_s_* and *c_t_* are *equivalent* iff *sim_0_*(*c_s_,c_t_*) ≥ 0.9.
2. *Related* (⊆, ⊇,⊓): Two concept elements *c_s_* and *c_t_* are *related* iff 0.9 > *sim*_0_ ≥ 0.5.
3. *Independent* (⊥): Two concept elements *c_s_* and *c_t_* are i*ndependent* iff 0.5 > *sim*_0_.

The similarity Equation (1) is used in a two-tier matching strategy to determine the mappings of two concept schemes explained in the next section.

#### 4.5.3. Two-Tier Matching Strategy

The process of concept alignment is based on a two-tier matching strategy that consists in two phases: element level matching and structure level matching.

The purpose of the process of concept alignment is to determine the mappings between two concept schemes at the granularity of concept elements. This means finding the alignment *A*′ given the pair of schemas *X* and *U* where *X* can be any concept scheme constructed from a profile supplier/consumer document and *U* will always be the ubiquitous user model concept scheme (as explained in 4.5.1). We want to determine the all the semantic relations *R* from the triplet <*c_s_,c_t_,R*> from all concepts in *X* to all concepts in *U* as shown in Equation (3):

(3) $$R\left(c_{s},c_{t}\right)\forall c_{s}\in C_{S},\forall c_{t}\in C_{T}$$

We are considering two types of links between two concepts of different concept schemes based on SKOS mapping properties that establish associative, and interchangeability mappings. A hierarchical mapping link between two concepts, established by the properties skos:broadMatch and skos:narrowMatch are not useful for this work since interchange and reuse is intended.The property skos:relatedMatch is used to state an associative mapping link between two concepts.

The most important mappings, for our purposes, are interchangeability mappings. The skos:closeMatch property states that the two linked concepts are “sufficiently similar that they can be used interchangeably in some information retrieval applications” [43] skos:exactMatch links two concepts, “indicating a high degree of confidence that the concepts can be used interchangeably across a wide range of information retrieval applications skos:exactMatch is a transitive property, and is a sub-property of skos:closeMatch” [43].

In the element level matching step, the concepts are directly compared to each other without considering the hierarchy structure and values. The goal of element level matching is given concept *c_s_* of the source concept scheme *X_s_*, finding the best concept label c*_tb_* from a set of concept candidates for alignment in the target concept scheme *X_T_*.

In the structure level matching step, the context of the source and target concepts (neighbors of the concepts in the hierarchy) are considered. The ultimate goal of this process is determine the one-to-one mappings between the concept *c_s_* of the source concept scheme *X_s_* and the best concept c*_tb_* from the set of labels *C_t_* of the target concept scheme *X_T_*. From this phase we can also obtain decision recommendations for the inclusion of new concepts, sub collections and collections in the ubiquitous user model concept scheme allowing it to evolve over time.

##### 4.5.3.1. Element Level Matching Phase

The element level matching phase provides reasoning on the similarity of the isolated concept element. From this stage we can obtain the relation *R_0_* of every triplet <*c_s_,c_t_,R_0_*> ∀*c_s_* ∈ *C_S_* ∀*c_t_* ∈ *C_T_* which is a first attempt in finding the semantic mapping relation if we consider only the concept labels without context. The element level matching phase consists in three steps:

1. Calculates the similarity matrix between *C_s_* and *C_T_* with equation 1. The concepts with the highest similarity scores in the target are considered the best suited candidates for alignment (*c_tb_*).
2. Determines determine the target collection to which the source document is most related, and to which sub collection each concept in the source *c_s_* belong. The most related collection is determined by calculating which target collection has the higher relative frequency of membership of best suited concepts [Equation (4)]:
  (4) $$r.f.\left({\text{colleccion}}_{i}\right)=\frac{f\left(c_{\text{bt}}\right)}{\text{count}\left(c_{\text{bt}}\right)}\forall i\in \text{DimensionsU}$$
3. Determines the relation *R_0_* between *c_s_* and *c_T_* according to the criteria presented in Section 4.5.2 given the similarity results of the similarity matrix calculated in step 1.

The mapping relations cannot only be determined with the element level matching because from this process we can have the following possible outcomes:

Case 1. Exactly one concept label of the target has *sim_0_* = *1*, the best suited concept is clearly defined, but it can be the case that the labels are homonymous.
Case 2. More than one concept label of the target has *sim_0_* = *1* or *equivalent* relation, so in order to select just one concept in the target for alignment context in the hierarchy must be considered.
Case 3. No concept label of the target is classified as *equivalent* and the best score is *related*.
Case 4. No concept label of the target is classified as *equivalent* and the best score is *independent*.

When it is the case 3 or 4, no exact match is found and we need to consider context in order to determine new additions of concept, sub collection or collection to the target scheme. For every case in the previous outcomes, reasoning on structure is needed.

##### 4.5.3.2. Structure Level Matching Phase

The structure level matching provides reasoning on structure in order to verify or decline the results obtained in the element level matching phase. This phase consists of three steps:

1. Select the sets of neighbors *N_s_* and *N_T_* from *H_s_* and *H_t_* which define the context of each concept in the source *c_s_*. The ancestors of *c_s_* and siblings that are terminal elements and share the same parent are considered for *N_s_*. The set of neighbors *N_T_* from the target hierarchy *H_t_* is populated with the labels of the target concepts the sub collection in which the best suited concept for alignment *c_tb_* is a member. If more than one *c_tb_* is chosen for *c_s_* in the previous element level matching, the labels of all corresponding sub collections are selected for the *N_T_* set. It is important to note that neither *c_s_* nor *c_tb_* are included in the sets of neighbors, with the aim of evaluating only the context of these concepts by itself.
2. Calculate a new similarity matrix between the two sets of neighbors *N_s_* and *N_T_* using similarity equation 1 determining $R_{1}\left(c_{s}^{\prime },{c^{\prime }}_{tb}\right)\forall c_{s}^{\prime }\in N_{S},\forall c_{tb}^{\prime }\in N_{T}$.
3. Given the highest relation *R_0_ (c_s_,c_tb_)*obtained from the element level matching phase and the highest relation $R_{1}\left(c_{s}^{\prime },{c^{\prime }}_{\text{tb}}\right)$ resulting of the structure level matching similarity calculation, the next step is apply IF THEN rules to determine the one-to-one mappings and decision recommendation for the inclusion of new concepts, sub collections and collections in the ubiquitous user model concept scheme allowing it to evolve over time.

### 4.6. Integration and Consumption of Profile Information

In order to integrate a ubiquitous user model gathering profile information from heterogeneous sources and share and reuse this information, two main processes are done: (i) integrate a profile supplier information to the ubiquitous user model (Algorithm 1) and (ii) consume profile information from the ubiquitous user model (Algorithm 2).

 **Algorithm 1.** Integration of profile supplier information to ubiquitous user model.**Require:** U2MIO ontology**Ensure:** All the semantic mappings *M^Xi,U^*, U2MIO enhance recommendations and new instance in U2MIO with profile information 1:Extract *sd_i_* from profile information supplier 2:Save *sd_i_* with a timestamp and source id in the Raw data repository 3:**if** source id is new to U2MIO **then** 4: create a new concept scheme *X_i_*, identify (*C_i_*, *H_i_*, *V_i_*) and insert an instance with *V_i_* values in U2MIO **comment:** Element level matching 5: Extract *C_T_* from the ubiquitous user model concept scheme *U* 6: Calculate the relation *R_0_* of every triplet 〈*c_s_*, *c_t_*, *R*_0_〉 ∀*c_s_* ∈ *C_S_*, ∀*c_t_* ∈ *C_T_* **comment:** Structure level matching 7: Select the set of neighbors *N_S_*, ∀*c_s_* ∈ *C_s_* and *N_T_*, ∀*c_tb_* ∈ *C_T_* 8: Calculate relation *R_1_* of every triplet 〈*c*′*_s_*, *c*′*_tb_*, *R*_1_〉 ∀*c*′*_s_* ∈ *N_s_*, ∀*c*′*_tb_* ∈ *N_t_* 9: Apply IF THEN rules given *R_0_* and *R*_1_ ∀*c_s_* ∈ *C_s_* and obtain *M^Xi,U^* = *map* (*c_s_*, *c_tb_*) ∀*c_s_* ∈ *C_S_*, ∀*c_tb_* ∈ *C_T_* and U2MIO 10: Add *M^Xi,U^* to U2MIO and implement recommendations 11:**else** 12: Update *X_i_* instance with *V_i_* values in U2MIO 13:**end if**

As a precondition of both algorithms, we require the U2MIO ontology which contains the current ubiquitous user model concept scheme, the concept schemes and mappings of previously integrated sources and their instances. We defined Algorithm 1 for the integration of profile supplier information to the ubiquitous user model. Algorithm 2 describes how to reuse information from other sources. Both algorithms use the process of concept alignment when the supplier or consumer is not known to define all the semantic mappings *M^Xi,U^* between a interoperability stakeholder concept scheme *X* and the ubiquitous user model concept scheme *U*.

It is important to notice that an instance with only the most recent values extracted from each sources are stored. The concept scheme of the ubiquitous user model allows only the addition of new concepts, sub collections and collections from the process of concept alignment recommendations.

Two statements from Algorithm 2 deserve further explanation. It is easy to deduce that it is likely that a required value can be populated from more than one source, so the query (statement 11) can get several requested value candidates.

 **Algorithm 2.** Consumption of profile information from the ubiquitous user model.**Require:** U2MIO ontology ∈**Ensure:** Profile consumer′s required values delivered 1:Receive *cd_i_* from profile information consumer request 2:**if** consumer id is new to U2MIO **then** 3: create a new concept scheme *X_i_*, identify (*C_i_*, *H_i_*, *V_i_*) and insert an instance with *V_i_* values in U2MIO **comment:** Element level matching 4: Extract *C_T_* from the ubiquitous user model concept scheme *U* 5: Calculate the relation *R_0_* of every triplet 〈*c_s_*, *c_t_*, *R*_0_〉 ∀*c_s_* ∈ *C_S_*, ∀*c_t_* ∈ *C_T_* **comment:** Structure level matching 6: Select the set of neighbors *N_S_*, ∀*c_s_* ∈ *C_s_* and *N_T_*, ∀*c_tb_* ∈ *C_T_* 7: Calculate relation *R_1_* of every triplet 〈*c*′*_s_*, *c*′*_tb_*, *R*_1_〉 ∀*c*′*_s_* ∈ *N_s_*, ∀*c*′*_tb_* ∈ *N_t_* 8: Apply IF THEN rules given *R_0_* and *R*_1_ ∀*c_s_* ∈ *C_s_* and obtain *M^Xi,U^* = *map* (*c_s_*, *c_tb_*) ∀*c_s_* ∈ *C_S_*, ∀*c_tb_* ∈ *C_T_* and U2MIO enhance recommendations 9: Add *M^Xi,U^* to U2MIO 10:**end if** 11:Get concept requested *V_i_* from concepts with *exactmatch* in U2MIO 12:Select V_i_* with conflict resolution process 13:Deliver V_i_* 14:**if** consumer is not also supplier 15: Save *cd_i_* with values, timestamp and consumer id in Service usage historical data repository 16:**else** 17: Save *cd_i_* with values, timestamp and consumer id in raw data repository 18:**end if**

A conflict resolution process must select the best suited value to deliver (statement 12). For the time being, the best value is selected taking only into account the effective date of extraction from the original source, delivering the most recent value.

## 5. Application Scenarios

According to the World Health Organization (WHO), overweight and obesity are serious public health challenges, and their prevalence is growing worldwide even in countries with low or middle income [44]. Mexico ranks second worldwide for prevalence of obesity, after the United States of America [45]. Overweight and obesity represent a public health problem identified as a priority for many countries, because of the economic and social costs involved. They are important risk factors for developing chronic diseases, including cardiovascular, diabetes and cancer. Other solutions have been proposed for these mayor related health problems like diabetes [46].

The World Health Organization defines overweight and obesity as an abnormal or excessive fat accumulation that presents health risks. If a person consumes more calories than he expends, he will gain weight, resulting in overweight and obesity. This is due to unbalanced diets and low physical activity. In order to quantify overweight, anthropometric indicators are used that consider weight and height. The main indicator is the body mass index (BMI) which is the ratio between the weight and the square of the height of a person. In the case of obesity, waist circumference is also used as an indicator. An adult with a BMI of 30 or more is considered obese. An adult with a BMI greater than or equal to 25 is considered overweight [47].

A major challenge for creating personalized health applications is to capture the information needed for personalization in a user-friendly way, and avoid having to repeat this task for each application. We considered an application scenario of sharing and reusing data of heterogeneous profile suppliers to enhance our ubiquitous user model and determine parameters of Web Services in order to deal with overweight and obesity. Our adaptive system will recollect the essential profile data to watch and control overweight and obesity from different profile suppliers interpret the information and store new data in the ubiquitous user model.

From social networks and FOAF, the system can obtain explicit information provided by the user like basic profile demographic data, food preferences and sport interest. There are specialized health sites and web applications to monitor a person's diet and physical activity keeping a record of daily observations of the person's meals and activity are needed, with the purpose of calculating the daily calorie balance between caloric intake and calories burned during exercise. These observations can be explicitly captured by the user in mobile devices, in web sites or even downloaded from accelerometers, pedometers and commercial sensors that track time, distance, pace and even calories burned during an exercise session.

Other important medical and health profile suppliers can be the Personal Health Record that contains vital information about medical and physical condition. All these data can be obtained in XML format or transformed to it. Once the information is stored in the ubiquitous user model, it can be used to enhance information of the suppliers and as parameters in Web services. For the task of monitoring the training activity there are a variety of personal gadgets with advanced sensors, coupled with specialized Web and social network applications. Other applications help monitoring the daily consumption of calories and provide means to build and follow a personalized diet plan.

Application scenario using the ubiquitous user model for dealing with overweight and obesity, exemplifies profile information suppliers providing static, semi-static and dynamic information about the user.

The demonstration in Section 6 shows how two important processes for the construction and manipulation of the flexible ubiquitous user model can be used to solve the interoperability between applications explained in the previous example.

The first process is defining one-to-one semantic mapping between the concepts from a new source and existing concepts in the ubiquitous user modeling structure. In the example we considered gathering information from Polar devices with sensors attached: a Polar RS300x watch in addition with a Polar S1 food pod. The XML document can be obtained directly from the devices or downloaded from the web application which is named “A” in the previous example description. This same process could define the mappings between source document concepts from a different profile supplier, for example a Facebook, and the ubiquitous user model concepts. Social network profiles contain useful data for dealing with overweight and obesity. In the first process, we established the link between new profile supplier information and the ubiquitous user model. If we want to share this information with a profile consumer we have to define the semantic mapping between the new profile consumer concepts and the ubiquitous user model concepts.

The second process explains defining one-to-one mappings between the best suited concepts of the ubiquitous user model and the input parameters of a Web Service of provider “B” to load information to its web site preventing the user from explicitly capturing it. This process allows enhancing “B” fitness application's user profile. It also proves how web services personalization can be done identifying useful concepts from the user model that can satisfy web service input parameters requirements. The demonstration described in Section 6, presents conditions and results of a model example.

## 6. Demonstration and Results

As we mentioned in previous section, in order to help the user dealing with overweight and obesity, it is important to monitor physical activity and training sessions. For our demonstration, we considered the case of gathering information about training sessions with a Polar RS300x watch in addition with a Polar S1 foot pod. With these devices we were able to get information about personal settings and running sessions. A Polar FlowLink device and WebSync software are required for data transfer to the polarpersonaltrainer.com web site. Afterwards an XML document was extracted from the web application. The information obtained by the source was interpreted and integrated this to the ubiquitous user model. Later, some data will be reused as preferred values in the use of a web service. A Trainingpeak web service [48] is considered as profile information consumer, as we seek to use the ubiquitous user model to adapt and personalize web services. This is a proof of concept experiment in order to test the integration of the ubiquitous user model gathering information from heterogeneous sources, the alignment process between similar concepts based in SKOS and the reuse of information for web service personalization.

In this case, credentials needed for authentication to both the profile supplier web site and the web service as profile consumer, were given explicitly by the user in order to avoid privacy issues. The automatic management of authentication is an issue for further research. The document extracted from the profile supplier is in XML format and contains information about several training sessions. Table 2 shows a view of source XML schema.

The adaptive system was programmed in C# and for the WordNet based similarity measure WordNet 2.1 was used as well as a modified version of [49]. The ubiquitous user model concept scheme was build reusing SKOS ontology and its classes as metaclasses to build an initial ubiquitous user model concept scheme with Protégé. New concepts will be added automatically by the adaptive system. In order to avoid cold start problem the ubiquitous user model structure was previously configured according to the dimensions explained in 4.1 and populated with domain concepts explicitly. Domain ontologies can be used for the automatic initial population. In this case the classes and hierarchy of the domain ontology chosen will turn in collections, sub collections and concepts in the ubiquitous user model.

**Figure 6.**
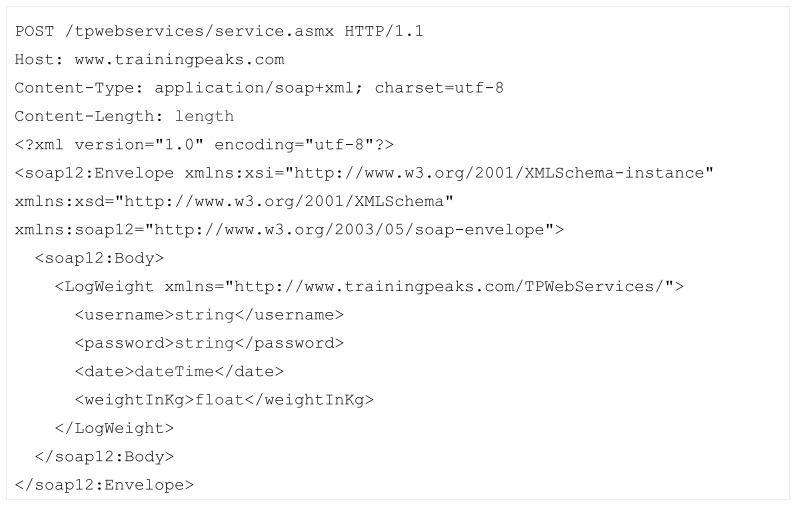
Sample SOAP 1.2 request of LogWeight web service.

As a profile consumer, we simulated a request to training peaks LogWeight web service. The main idea is to automatically replace the actual values of the parameters needed (other than authentication credentials for this experiment) for the web service SOAP request shown in Figure 6 [48].

The placeholders are presented in blue. In this case, the system automatically must place date and *weightInKg* parameters.

We divided the results of the demonstration in two subsections. In 6.1, we present the results of mapping the concepts from a new source to existing concepts in ubiquitous user model. We show the results of personalizing a web service reusing information with the mediation the ubiquitous user model in 6.2.

### 6.1. Results of Mapping the Concepts from a New Source to Existing Concepts in the Ubiquitous User Model

First of all the system preprocess the source XML document to extract the labels of the terminal elements (elements which directly contain a value) where each label is a *c_s_*∈*C_s_*. On the other side, the system takes into account all the concept labels consisting of lexical labeling, notation and documentation SKOS properties. Each label is a *c_t_*∈*C_t_*. To maintain the readability of the example, for the XML source document, a sample of the first concept labels of the XML document and a partial view of relevant skos:prefLabels of the ubiquitous user model are shown in the results. In the entire experiment all concepts of XML document and all types of SKOS lexical labels are considered.

The interoperability engine processes these concepts building a similarity matrix with the element level matching process described in 4.5.2. We present a partial view of similarity matrix in Table 3 showing just the most relevant concepts and collections. The first column represents eleven concepts of the source document that are *c_s_*∈*C_s_*.

The first row of Table 3 shows the skos:Collection names, in this case, Demographic data and general user data and Observations & Measurements. Each collection has several sub collections whose concepts are presented in the second row of Table 3. Only the most relevant collections and sub collections for the experiment and a sample of most relevant concepts are presented for readability. Each cell represents a result of the calculation of *sim_0_(c_s_,c_t_)* described in Equation (1). Although the similarity measures are in fact categorized with criteria to determine the relation presented in 4.5.2, we chose to show the actual similarity values in Table 3.

Some interesting similarity measures are highlighted for its analysis. If the cell is highlighted in red, it means that the concept label from the source is not expressive enough, so further disambiguation must be done in structure level matching process. A new concept might be added. If the cell is highlighted in yellow, this means that no clear *equivalence* is found so far; it is a possible problem for this match. For instance, the similarity of *name vs. name* seems equivalent but they are homonymous, because in the source it is the name of the exercise and in the target it refers to the person's name. Further disambiguation must be done in structure level matching process.

The same stands for *calories*. In this case, we obtained two target exact matches and they refer to calories burned in exercise and daily calorie intake, but it is impossible to tell the difference with element level process. For the concept *duration*, we obtained that two source concepts can be *equivalent* to the same target concept. If the cell is highlighted in green, it means that the *equivalence* is very likely to be correct.

From element level matching results the most related collection is calculated with Equation (3). Only the most relevant for the experiment collections of the ubiquitous user model are presented in Table 3: {*Demographic data and general user data, Observations & measurements*}. The observations & measurements collection has three sub collections: {*Health O&M, Physical activity & training, Food &Diet*}. The collection with higher relative frequency of membership of best suited concepts is the *Observations & measurements* collection.

We present, in detail, interesting results of some cases found in element level matching process. They correspond to the matching process for concepts *name*, *calories* and *weight*.

In the first step of structure level matching process, sets of neighbors are generated as described in Section 4.5.3. For the set of neighbors *N_s_* from the source XML document hierarchy *H_s_*, the concept labels of the ancestors of *c_s_* and direct brother who share the same immediate parent element and are terminal elements are selected.

The set of neighbors *N_T_* from the ubiquitous user model hierarchy *H_t_* is populated with the labels of the target concepts the sub collection in which the best suited concept for alignment *c_tb_* is a member.

A sample of the sets of neighbors generated in structure level matching process is presented in Table 4. Notice that the labels of the brother concepts are concatenated with a prefix of the direct parent. With this strategy, we are aiming to add more expressivity to the labels of commonly used generic labels.

Once the sets of neighbors are generated, the system processes proceeds to the calculation of a new similarity matrix, based in element level matching and equation 4, between the two sets of neighbors *N_s_* and *N_T_*. Tables 5–7 are the similarity matrixes for pair of sets of neighbors (*N_s_,N_T_)* for the concepts *name*, *calories* and *weight* in that order. As it was expected, Table 5 shows no high similarity of the neighbors of concept name because *c_s_* and *c_bt_* are homonymous.

As we recall, from the process of element level matching for *calories* concept (Table 3), two best suited concepts in different sub collections were found. Therefore the neighbor set *N_T_* included concepts from both sub collections. This strategy helps disambiguate the sense of the concept of the source by analyzing its similarity with the concepts of the two sub collections in conflict. In the case of the concept calories the sense was clearly disambiguated.

Table 6 shows how to equivalences were found in the neighbors' similarity matrix and both of them belong to the same sub collection: the sub collection corresponding to physical activity and training domain. These means that the sense of extracted data from the source, corresponds to calories burned in the domain of physical activity instead of intake of calories, in the domain of food and diet.

The similarity matrix of the concept *weight* described in Table 7 shows that one concept *equivalence* was encountered in the same sub collection as the *equivalence* found in the element level matching process. This means that weight from the source is classified in the correct domain and sub collection (*Health O&M*) and therefore both concepts are not homonymous.

The third step of the structure level matching is applying a set of IF THEN rules to determine the one-to-one mappings between the concept *c_s_* of the source concept scheme *X_s_* and the best concept *c_tb_* from the set of labels *C_t_* of the target concept scheme *X_T_*.

In Table 8 we present the results of both processes, element and structure level matching, and the final mapping determined for *MAP*(*c_s_,c_tb_*) after the process of rule application.

As we stated in rule 3, adding a new concept in the most relevant collection for *name* can be considered but the label of *c_s_* must be concatenated to the immediate ancestor label for disambiguation. This new label for the skos:Concept would be *exercise_name*, which is more expressive of the real sense of the concept.

The process of structure level matching successfully determined the right match for *calories*, despite the resulting conflict of the element level matching. Likewise, the mapping for concept *weight* was successful.

### 6.2. Results of Personalizing a Web Service Reusing Information with the Mediation of the Ubiquitous User Model

The same process of concept alignment for interoperability used for mapping the concepts from a new source to existing concepts in ubiquitous user model is functional for interoperability with web services. First of all, the concept labels of the SOAP web service request are selected as concepts ***c****_s_***∈*C****_s_*. It is important to notice that for this experiment authentication parameters were not considered.

For the target side, a selection of relevant skos:prefLabels of the ubiquitous user model are used as concepts ***c****_t_***∈*C****_T_*.

The similarity matrix is calculated with the web service request concepts and the ubiquitous user model concepts in the element level matching, is analogous to Table 3, which was built for the source XML document. In Table 9, we present this similarity matrix. It shows that for the concept *date* three best suited concepts for alignment were found. Date is a concept that is almost always attached to different kind of observations and measurements, since the nature of this information is dynamic. Once again, structure level matching will help to determine the sub collection of membership of the concept *date*. For the concept *weightInKg*, one *equivalence* is found and the best suited concept for alignment in the target side is *weight*.

Since all the resulting best suited concepts for alignment are in the same collection, to determine the most related collection is fairly straightforward, and it correspond to *Observations &measurement*.

The structure level matching process is also followed as described in Section 4.5.3. Neighbor sets are generated for each *c_s_* and new similarity matrixes are computed for each pair (*N_s_,N_T_)*. Table 10 shows the similarity matrix for the sets of neighbors of *date* concept and Table 11 is the analogous matrix for concept *weightInKg*.

From Table 10 and Table 11 we can conclude that, in both cases, the concepts *date* and *weight* belong to the same sub collection: *Health O&M*. Although it is difficult to determine just examining the labels, that *date* concept corresponds to this collection, the structure level matching strategy establish without a doubt, the membership to *Health O&M*.

The application of IF THEN rules is the next step of structure level matching. We applied the first rule in both cases, as it is shown in Table 12. This means that the concepts of *date* and *weightInKg* and ultimately the corresponding parameters of the web service request, have an exactMatch with the concepts in ubiquitous user model. These one-to-one mappings establish interoperability between the web service and the ubiquitous user model enabling sharing and reuse with other sources with the mediation of the user model.

In this experiment we established a link between profile suppliers and profile consumers at concept level, but we cannot be sure that the values are completely interchangeable. We established an exact match between the concept *weight* in polarpersonaltrainer.com application with the concept *weightInKg* from Trainingpeaks loaded to the application via the web service, but at this point we cannot know if further transformations are needed. It is possible that the units of measurement are not the same, for instance. Further research must to be done in this area.

### 6.3. Summary Results of Schema Matching Process

We presented in Section 6.1 the integration of the training sessions gathered with a Polar RS300X watch in addition with a Polar S1 foot pod, to the ubiquitous user model. The XML document of the training sessions mentioned was modeled with a concept scheme *P* containing 31 source concepts to be aligned with the concepts of the target ubiquitous user model concept scheme *U*. The outcomes were evaluated by a human expert who decided if the semantic relations found were correct and recommendations make sense. The outcomes of the matching process done for the alignment of the concepts in the concept schemes *P* and *U* are shown in Table 13. The best exactMatch semantic relations were found for nine concepts. The best suited concept for alignment in the target concept scheme for four concepts was not found.

This error is due to the use of abbreviations in the source XML document (for example *avg* instead of *average*). This problem can be solved using alternate and hidden SKOS labels in the target concept scheme, but this knowledge must be provided by a human expert or domain ontology. All recommendations given by the process was described as reasonable by the expert. No closeMatch relation was found.

After the integration of the previous mentioned source document, we try to reuse some of the information of this profile supplier to personalize a Web service of provider “B”, in this case LogWeight Web Service from Training peaks. A concept scheme *W* was built with the concept labels of the SOAP web service request parameters, in this case *date* and *weight*. It is important to notice that for this experiment authentication parameters were not considered. The outcomes of the matching process done for the alignment of the concepts in the concept schemes *W* and *U* are shown in Table 14:

With this toy example, we showed that enrichment of a fitness application and web service personalization was possible with our proposed solution. The results with this toy example only proof the feasibility of our solution.

## 7. Discussion

Berkovsky *et al.* [10] suggest in their future research directions the use of domain thesauri and WordNet to identify synonyms; develop flexible mechanism that allow applying a central ontology in different domains. The authors say that these research directions “may help bridging the gap between the two approaches” described above.

Our contribution is the proposal of a flexible ubiquitous user model representation that adapts itself dynamically to new user modeling interoperability actors, and a strategy to automatically define mappings between concepts to enable data exchange. This approach deals with syntactic and semantic heterogeneity with the least intervention of profile suppliers and consumers.

Our approach differs from standardization-based user model ontologies like GUMO [3] in which user modeling interoperability participants have to adhere to syntactic and semantic standards. We provide flexibility to the central ubiquitous user model enabling it to change, and relieve the profile suppliers and consumers from adhering to format or semantic standards. We consider our solution as a mixed approach [9] where there are scattered local storages of user data from each profile provider which refer to a shared ubiquitous user model in a conceptually centralized perspective.Systems based on user profile management and selection like [36] aggregate different versions of user profile for each service and automatically select the best suited version for the user current state. The same information with minor changes is saved several times for each service, causing redundancy and inconsistencies between the user models of heterogeneous sources. In our ubiquitous user model just one version of the user profile information of a source is stored and information can be reused at concept granularity avoiding redundancy.

In other mediation based user modeling systems [29] mappings are made by human effort making it hard to include new participants in user modeling interoperability. In [7] the authors presents a highly flexible system, but to take part in the interoperability process, every provider need to comply to a standard format for the exchange and maintain a sharable user model which includes the fragments of user model as RDF statements. User modeling systems must do the effort of maintaining a sharable user model in a standard format. In our approach, the effort of syntactic and semantic interoperability is made by the user-adaptive system.

## 8. Conclusions

We have presented a first attempt to build a ubiquitous user model for profile sharing and reusing based on a flexible user profile structure that can evolve in time and an automated process of concept alignment. The ubiquitous user model classification scheme is build using SKOS as a common data model to enable data sharing between profile suppliers end profile consumers. We proposed a distributed architecture for the adaptive system in order to exchange and reuse information from heterogeneous sources: social networking applications, microformats and personal devices with sensors.

A two-tier matching strategy for concept schemas alignment was proposed. Additionally, we showed that the process of concept alignment for interoperability based in an element level matching and a structure level matching can allow the interoperability between heterogeneous sources. Demonstration was made in the application scenario of sharing and reusing data of profile suppliers to enhance our ubiquitous user model, mapping the concepts from a new source to existing concepts in ubiquitous user model and personalizing a web service in order to deal with overweight and obesity. Application scenario using the ubiquitous user model for dealing with overweight and obesity, exemplifies profile information suppliers providing static, semi-static and dynamic information about the user. For our experiments, we considered gathering information about personal settings and running sessions from commercial devices with sensors, and personalize a web service reusing information with the mediation of the ubiquitous user model. Credentials needed for authentication to the profile supplier web site and the web service as profile consumer, were given explicitly by the user in order to avoid privacy issues. We are accessing profile information from different sources on the basis that the user provides the authentication credentials. This means that the user is identified and allows such information transfer. Hence mechanisms for preserving privacy of the ubiquitous user model information should be developed.

Our results show that the automated concept alignment process found one-to-one mappings between source and target concepts and suggested the insertion of new concepts to the ubiquitous user model except when abbreviations were used in the source document. The addition of new concepts suggested by this same process, allows the evolution in time of the ubiquitous user model. The recommendations resulting of the matching process were qualified as reasonable by a human expert. The alignment process was able to provide suited preferred values for the web service request. These results are encouraging because they show that it is possible to automatically find and establish the proper semantic relations between concepts of various actors of user modeling interoperability with the process of concept alignment proposed.

Although exact matches were established at concept level, we cannot know if further transformation is needed. It is possible that value transformation may be needed in order to find a perfect equivalence between concepts and values. Further research must to be done in this area. We still have to work on the formalization and validation of the proposed approach with more intensive design experiments with focus groups.

## Figures and Tables

**Table 1.** Used matching techniques.

**Type of similarity**	**Purpose**	**Similarity measure**
String similarity	High lexical similarity	Dice coefficient
	Label inclusion	Longest common substring [Equation (2)]
Semantic similarity	Label equivalence	WordNet

**Table 2.** Partial view of source XML schema.

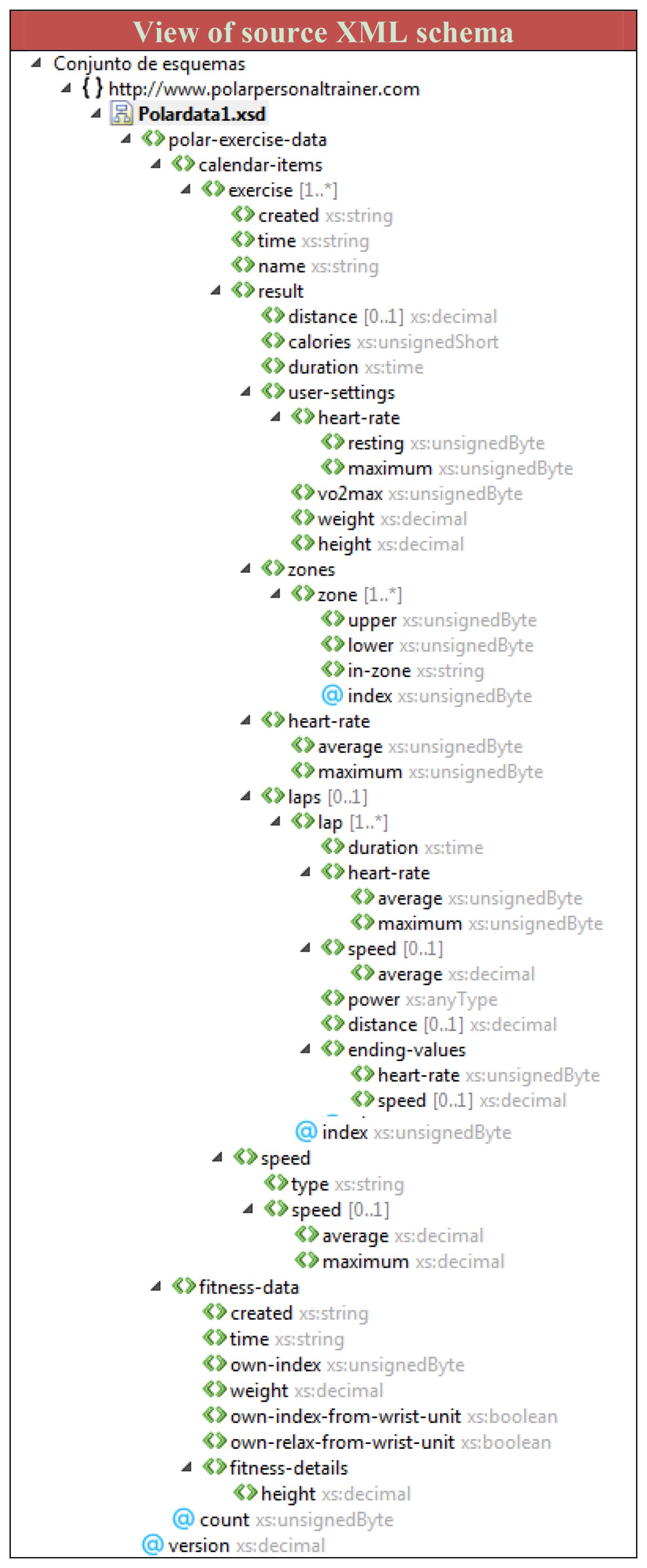

**Table 3.** Partial view of similarity matrix (XML Document concepts, ubiquitous user model concepts).

**u2m**	**Demographic data and general user data**			**Observations & measurements**															
				**Health O&M**			**Physical activity & training observations**									**Nutritional Observations**			
**Source**	**Name**	**Age**	**Gender**	**Date**	**Weight**	**Height**	**Start Date**	**End Date**	**Training sesion**	**Duration**	**Distance**	**HR Acg**	**Calories**	**Pace Avg**	**Speed Avg**	**Calories**	**Fat**	**Carbs**	**Protein**
created	0.5	0.33	0.29	**0.75**	0.17	0.17	0.59	0.43	0.21	0.29	0.14	0.17	0.25	0.31	0.33	0.25	0.67	0.29	0.29
time	0.5	0.86	0.62	0.83	0.67	0.88	0.81	0.81	0.50	**0.91**	0.77	0.41	0.53	0.50	0.41	0.53	0.67	0	0.25
name	**1**	0.5	0.4	0.57	0.46	0.59	0.53	0.60	0.4	0.46	0.46	0.39	0.4	0.46	0.46	0.4	0.43	0.25	0.29
distance	0.46	0.71	0.62	0.83	0.67	0.89	0.83	0.83	0.44	0.62	**1**	0.53	0.57	0.49	0.45	0.57	0.67	0.2	0.31
calories	0.4	0.53	0.38	0.57	0.8	0.4	0.57	0.57	0.31	0.53	0.57	0.42	**1**	0.42	0.31	**1**	0.4	0.5	0.14
duration	0.46	0.86	0.67	0.77	0.71	0.71	0.72	0.74	0.54	**1**	0.67	0.45	0.53	0.61	0.57	0.53	0.71	0.2	0.38
resting	0.25	0.33	0.17	0.25	0.22	0.17	0.38	0.38	**0.52**	0.29	0.38	0.17	0.29	0.14	0.14	0.29	0.33	0.2	0.29
maximum	0.43	0.67	0.59	0.62	0.62	0.71	0.62	**0.74**	0.38	0.57	0.71	0.46	0.53	0.56	0.44	0.53	0.62	0.2	0.27
vo2max	0.25	0.33	0	0.25	0	0	0.17	0.17	0.17	0.17	0.17	0	0.17	0.17	0.17	0.17	**0.33**	0.2	0.17
weight	0.46	0.77	0.67	0.67	**1**	0.83	0.67	0.67	0.44	0.71	0.67	0.50	0.8	0.53	0.50	0.8	0.71	0	0.33
height	0.59	0.77	0.8	0.57	0.83	**1**	0.53	0.70	0.44	0.71	0.89	0.36	0.4	0.53	0.50	0.4	0.86	0	0.33

**Table 4.** Sample of sets of neighbors generated in structure level matching process.

*c_s_*	**Set of neighbors** *N_s_*	**Set of neighbors** *N_T_*
**name**	*{polar-exercise-data, calendar-items, exercise, exercise_created, excercise_time}*	*{age, gender}*
**calories**	*{polar-exercise-data, calendar-items, exercise, result, result_duration, result_distance}*	*{startDate, endDate, training_sesion, Duration, Distance, HR Acg, Pace Avg, Speed Avg, Fat, Carbs, Protein}*
**weight**	*{ polar-exercise-data, calendar-items,exercise, result, user-settings, user-settings_height}*	*{date, height}*

**Table 5.** Similarity matrix for pair of sets of neighbors (*N_s_,N_T_)* of concept *name*.

**Name**	**Age**	**Gender**
**polar-exercise-data**	0.3575	0.4925
**calendar-items**	0.4766667	0.5
**excercise**	0.43	0.38
**exercise_created**	0.3833334	0.35
**excercise_time**	0.61	0.4866667

**Table 6.** Similarity matrix for pair of sets of neighbors (*N_s_,N_T_)* of concept *calories*.

**Calories**	**Start Date**	**End Date**	**Training _sesion**	**Duration**	**Distance**	**HR Acg**	**Pace Avg**	**Speed Avg**	**Fat**	**Carbs**	**Protein**
polar-exercise-data	0.668	0.612	0.518	0.3875	0.3875	0.376	0.43	0.41	0.666666	0.4	0.215
calendar-items	0.6425	0.73	0.395	0.4766	0.51	0.3875	0.4475	0.44	0.44	0.4	0.28571
excercise	0.69	0.663	0.71	0.43	0.43	0.326	0.4	0.44666	0.4	0.25	0.14285
result	0.6533	0.74	0.523333	0.46	0.46	0.306	0.3066	0.373333	0.43	0.2	0.33
result_duration	0.76	0.825	0.56	**1**	0.6	0.455	0.575	0.57	0.666666	0.2	0.36333
result_distance	0.8099	0.855	0.525	0.5666	**1**	0.512	0.48	0.48	0.59	0.2	0.32333

**Table 7.** Similarity matrix for pair of sets of neighbors (*N_s_,N_T_)* of concept *weight*.

**Weight**	Date	Height
**polar-exercise-data**	0.75	0.33
**calendar-items**	0.5	0.44
**excercise**	0.43	0.4
**result**	0.46	0.43
**user-settings**	0.623	0.503
**user-settings_height**	0.61	**1**

**Table 8.** Mappings determined after rule application.

*c_s_*	**Rule applied**	**Element level matching result**	**Structure level matching result**	*MAP*(*c_s_,c_tb_*)
**name**	3	*Relation(c_s_,c_tb_) is equivalence and sim_0_*=*1*	*Relation(c*′*_s_*,*c*′*_tb_) is related and is not member of O&M collection*	Homonymous (no mapping)

**calories**	1	*Relation(c_s_,c_tb_) is equivalence and sim_0_*=*1 in {Physical activity & training} and sim_0_*=*1 in {Food &Diet}*	*Relation(c*′*_s_*,*c*′*_tb_) is equivalence in {Physical activity & training}*	*MAP*(*c_s_,c_tb_*) determined as skos:exactMatch.

**weight**	1	*Relation(c_s_,c_tb_) is equivalence and sim_0_*=*1 in {Health O&M}*	*Relation(c*′*_s_*,*c*′*_tb_) is equivalence in {Health O&M}*	*MAP*(*c_s_,c_tb_*) determined as skos:exactMatch.

**Table 9.** Partial view of similarity matrix (Web service request concepts, ubiquitous user model concepts).

u2m	Observations & measurements											
Web service	Date	Weight	Height	Start Date	End Date	Training sesion	Dura-tion	Dis-tance	HR Acg	Calo-ries	Pace Avg	Speed Avg
**date**	**1**	0.67	0.57	**1**	**1**	0.38	0.77	0.83	0.60	0.57	0.53	0.39
**weigthInKg**	0.25	**1**	0.83	0.64	0.64	0.42	0.64	0.62	0.55	0.77	0.57	0.512

**Table 10.** Similarity matrix for the sets of neighbors of *date*.

**Date**	Weight	Height	Training_sesion	Duration	Distance	HR Acg	Calories	Pace Avg	Speed Avg
**LogWeight**	**1**	0.833	0.111	0.125	0.125	0.166	0.25	0.125	0.1111
**weigthInKg**	**1**	0.833	0.266	0.25	0.3	0.166	0.125	0.125	0.1111

**Table 11.** Similarity matrix for the sets of neighbors of *weight*.

**weight**	Date	Height
**LogWeight**	0.25	0.8333
**date**	**1**	0.57

**Table 12.** Mappings determined after rule application.

*c_s_*	**Rule applied**	**Element level matching result**	**Structure level matching result**	*MAP*(*c_s_,c_tb_*)
**date**	1	*Relation(c_s_,c_tb_) is equivalence and sim_0_*=*11 in {Physical activity & training} and sim_0_*=*1 in {Health O&M}*	*Relation(c*′*_s_*,*c*′*_tb_) is equivalence in {Health O&M}*	*MAP*(*c_s_,c_tb_*) determined as skos:exactMatch

**weightInKg**	1	*Relation(c_s_,c_tb_) is equivalence and sim_0_*=*1 and sim_0_*=*1 in {Health O&M}*	*Relation(c*′*_s_*,*c*′*_tb_) is equivalence in {Health O&M}*	*MAP*(*c_s_,c_tb_*) determined as skos:exactMatch.

**Table 13.** Summary results of the matching process between *P* and *U*.

**Outcome**	**Right**	**Wrong**
exact Match relation found	9	
Concept discarded	9	**1**
Concept addition recommendation	4	**3**
New collection recommendation	3	
New sub collection recommendation	2	

**Table 14.** Summary results of the matching process between *W* and *U*.

**Outcome**	**Right**	**Wrong**
exactMatch	2	
